# Retrovirus mediated hypoxia-responsive element-regulated neurotrophin-3 transduction attenuates brain injury following focal cerebral ischemia in rats

**DOI:** 10.1186/1750-1326-7-S1-S16

**Published:** 2012-02-07

**Authors:** Junfeng Zhang, Qindong Shi, Pengbo Yang, Xi Xu, Xinlin Chen, Cunfang Qi, Jianshui Zhang, Haixia Lu, Pengbo Zhang, Bingqiao Zhao, Ping Zheng, Yong Liu

**Affiliations:** 1 Institute of Neurobiology, Xi’an Jiaotong University College of Medicine, #76 Yanta West Road, Xi’an, Shaanxi 710061, PR China; 2 The State Key Laboratory of Medical Neurobiology, Shanghai, 200032, PR China

## Background

Exogenous delivery of Neurotrophin-3 (NT-3) gene may provide a potential therapeutic strategy for ischemic stroke. But uncontrolled expression of NT-3 may cause deleterious side effects. Recently, hypoxia-specific gene expression systems have been developed in various ischemic diseases. To explore ischemia/hypoxia-controlled expression of NT-3 in rats, we constructed a recombinant retrovirus vector with 5HRE and NT-3 and delivered it to rat brain to investigate the neuroprotective effects of hypoxia induced NT-3 overexpression on focal cerebral ischemia.

## Methods

Three groups of rats received RV-5H-NT3, RV-5H-EGFP or saline injection, respectively. 3 days after gene transfer, the rats underwent 90 minutes of transient middle cerebral artery occlusion (tMCAO) and followed by 1 to 14 days reperfusion. Expression of NT-3 was detected by immunohistochemical staining and Western blot; neurological function was assessed by sensorimotor behavioral tests; infarct volume was determined by TTC staining; neuronal injury was examined by TUNEL.

## Results

NT-3 expression was significantly increased in RV-5H-NT3 transduced rat brain compared with RV-5H-EGFP or saline group 3 days after tMCAO (P < 0.05). Infarct volume was smaller in RV-5H-NT3 transduced rat brain than RV-5H-EGFP or saline group (P < 0.05) with reduced percentage of TUNEL positive cells (P < 0.05). Furthermore, functional recovery in RV-5H-NT3 transduced rats was better than RV-5H-EGFP or saline transduced group from 1 day to 2 weeks after tMCAO (P < 0.05).

## Conclusions

After RV-5H-NT3 gene transfer, NT-3 expression was up-regulated by five copies of HRE in response to hypoxia/ischemia, and hypoxia-regulated NT-3 expression attenuates ischemic brain injury and promotes functional recovery in cerebral ischemia rats.

